# Molecular dynamics analysis of a docking-derived baicalein–PPARγ ligand-binding-domain model suggests a pocket-associated AF-2-anchor-silent ensemble

**DOI:** 10.3389/fmolb.2026.1870075

**Published:** 2026-08-17

**Authors:** Xingyuan Kou, Ping Wu, Zhuo Liu, Jing Yan, Le Li

**Affiliations:** 1 Department of Radiology, Jianyang People’s Hospital, Chengdu, Sichuan, China; 2 Department of Pharmacy, Changde Hospital, Xiangya School of Medicine, Central South University, The First People’s Hospital of Changde City, Changde, Hunan, China; 3 College of Traditional Chinese Medicine, Changsha Medical University, Changsha, Hunan, China; 4 Department of Anatomy, Youjiang Medical University for Nationalities, Baise, Guangxi, China; 5 Department of Cardiology, The Affiliated Hospital of Southwest Medical University, Southwest Medical University, Luzhou, Sichuan, China

**Keywords:** activation function-2, baicalein, helix 12, MM/GBSA, molecular dynamics, peroxisome proliferator-activated receptor γ, ProLIF, Tyr473

## Abstract

Baicalein was examined in a selected docking-derived peroxisome proliferator-activated receptor γ (PPARγ) ligand-binding-domain (LBD) model to test whether pocket association, local helix 12 (H12) organization, and canonical activation function-2 (AF-2) anchoring were coupled under the sampled simulation conditions. Because no baicalein-bound PPARγ cocrystal structure was used, the results are interpreted as a selected-pose molecular dynamics model rather than as a definitive experimental binding mode. Three independent 100-ns replicas were analyzed with bound-state filtering, H12 and AF-2 descriptors, arm-resolved energetic decomposition, interaction fingerprints, a local Tyr473-engagement potential of mean force (PMF), and exploratory state annotation. Baicalein remained spatially associated with the ligand-binding pocket by the minimum-distance and center-of-mass criteria, whereas pocket-shell contact persistence was replica-dependent, with retained fractions of 94.20%, 94.00%, and 71.64% in replicas 1–3. Within bound-state-filtered frames and under the selected neutral baicalein/HIE histidine model, direct ligand hydrogen bonds to Ser289, His323, His449, and Tyr473 were not detected, and the AF-2 anchor-panel union occupancy was 0.00%. This anchor-negative pattern occurred despite measurable local H12 order, indicating that local H12 structural persistence and canonical AF-2 anchor assembly were separable in the selected model. Arm-resolved molecular mechanics/generalized Born surface area (MM/GBSA) descriptors and ProLIF fingerprints supported mixed Arm II/Arm III participation, with recurrent van der Waals contacts involving Cys285, Ile341, Leu330, Leu333, Arg288, and Ile326. The local Tyr473 PMF favored a displaced baicalein geometry, with a minimum at 0.66 nm along the Tyr473 hydroxyl-to-ligand-acceptor coordinate. Limited ligand-protonation and His323/His449-tautomer sensitivity screens did not recover persistent canonical AF-2 anchoring under the tested short-screen conditions. Exploratory state analysis resolved in-pocket heterogeneity but did not identify a descriptor-supported state with persistent canonical AF-2 anchoring. These simulations support a constrained structural model in which baicalein samples a pocket-associated, measurably H12-organized, anchor-negative PPARγ ensemble stabilized by non-canonical pocket interactions. The interpretation is limited to simulation descriptors and does not establish receptor activation, partial agonism, phosphorylation regulation, or transcriptional output.

## 1 Introduction

Peroxisome proliferator-activated receptor γ (PPARγ) is a ligand-regulated nuclear receptor in which the ligand-binding-domain links small-molecule recognition to conformational organization of the activation function-2 (AF-2) surface. In the classical full-agonist model, ligand-binding stabilizes helix 12 (H12) and promotes a coactivator-compatible AF-2 architecture. This state is commonly associated with a canonical anchor network involving Ser289, His323, His449, and Tyr473, with Tyr473 serving as a local reporter of agonist-like AF-2 engagement (Ahmadian et al., 2013; Tontonoz and Spiegelman, 2008; Choi et al., 2010; Choi et al., 2011).

PPARγ ligand behavior, however, is not fully captured by a binary distinction between ordered and disordered H12 states. Different ligand chemotypes and binding geometries can alter pocket interactions, H12 organization, and AF-2 anchoring in non-equivalent ways (Kroker and Bruning, 2015; Nolte et al., 1998; Bruning et al., 2007; Porskjær Christensen and Bahij El-Houri, 2018). A ligand may remain associated with the ligand-binding pocket and preserve measurable local H12 order without forming the Ser289/His323/His449/Tyr473 hydrogen-bond network observed in full-agonist references. Conversely, loss of a canonical AF-2 anchor signal does not by itself establish ligand dissociation or global H12 collapse. For this reason, ligand-retention, local H12 order, AF-2 anchor geometry, and subpocket interaction patterns need to be evaluated as separable descriptors.

Baicalein is a flavone ligand candidate (Tuli et al., 2020) with reported PPARγ-related activity in metabolic and bone-associated contexts. A recent type 2 diabetic osteoporosis (T2DOP) study reported that baicalein improved bone microarchitecture, reduced marrow adiposity, promoted osteogenic differentiation, suppressed adipogenic differentiation, and supported PPARγ–LBD (ligand-binding domain) engagement by docking, cellular thermal shift assay (CETSA), and molecular dynamics analyses (Zhang et al., 2026). Baicalein has also been reported to regulate macrophage lipid accumulation and inflammatory responses through the PPARγ/liver X receptor α (LXRα) pathway (Zhang et al., 2022). Because no experimentally resolved baicalein-bound PPARγ complex was used here, baicalein was analyzed from a docking-derived PPARγ–LBD starting-pose. This creates a specific interpretive requirement. Pose relaxation during unrestrained molecular dynamics must be distinguished from ligand dissociation, and ligand-dependent mechanistic descriptors should be interpreted only after explicit bound-state quality control.

Here, we used replicated molecular dynamics and descriptor-based analyses to determine whether baicalein samples a full-agonist-like AF-2 anchor state or a non-canonical pocket-associated ensemble. Baicalein was compared with full-agonist references, docked controls, non-canonical crystallographic references, and apo PPARγ–LBD. Three independent 100-ns production replicas were generated for each system, and baicalein was analyzed through a common 10–90 ns parent window with bound-state filtering where required.

The analysis addressed three related questions. First, we tested whether baicalein remains associated with the ligand-binding pocket after relaxation from the docking-derived pose. Second, we examined whether local H12 organization is coupled to direct AF-2 anchor hydrogen bonding. Third, we evaluated whether baicalein recognition is organized around Tyr473-centered anchoring or through alternative subpocket interactions. To address these questions, we combined ligand-retention quality control, reference-state atlas projection, H12 secondary-structure and local-order metrics, geometric AF-2 anchor analysis, pocket-H12 coupling, arm-resolved MM/GBSA decomposition, ProLIF interaction fingerprints, a local Tyr473-engagement potential of mean force, and exploratory state annotation.

The study was designed to characterize the structural and dynamic behaviors of the selected docking-derived baicalein–PPARγ–LBD model under the stated simulation, chemistry-state, and descriptor conditions. It was not designed to infer directly transcriptional activation, partial agonism, or cellular signaling. By treating H12 order, AF-2 anchoring, Tyr473 engagement, and arm-level pocket stabilization as distinct descriptors, the analysis provides a constrained framework for comparing the selected baicalein model with full-agonist and non-canonical reference systems and for defining testable hypotheses for future full-length alternative-pose simulations, broader chemistry-state calculations, and experimental studies.

## 2 Materials and methods

### 2.1 Structural models and system construction

The comparison set comprised nine independently prepared PPARγ–LBD systems: eight ligand-bound systems and one apo reference. Baicalein was the target ligand-bound system and was analyzed from a docking-derived starting-pose because a baicalein-bound PPARγ cocrystal structure was not used in this study. The baicalein docking route used the PPARγ–LBD receptor scaffold prepared from the 4YT1 chain A background after removal of the crystallographic ligand and non-protein heteroatoms. 4YT1 chain A was chosen as the scaffold because it provides a high-resolution PPARγ–LBD structure with an open ligand-binding pocket and uses the same construct numbering as the partial-agonist/JJB reference systems in the comparator panel. Baicalein was therefore treated as a proposed binding pose on a receptor scaffold rather than as an experimentally resolved baicalein complex. JJB_native was prepared from the MEKT76 (Protein Data Bank ligand code JJB) JJB/MEKT-76 ligand class, and JJB_dock was prepared from the matched redocked control. Full-agonist references included DAR_native (darglitazone cocrystal route), DAR_dock (matched redocked darglitazone control), and rosiglitazone (ROSI) (rosiglitazone cocrystal route). Amorfrutin 1 (2YFE) and piperine (9L3T) were used as non-canonical crystallographic references.

Each system was prepared from its own starting structure rather than from a shared receptor template. Docked controls and target docking-derived systems (DAR_dock, JJB_dock, and baicalein) were used to test the sensitivity of the descriptor atlas and state annotation to starting-pose variation relative to crystallographic references and were not treated as additional experimentally resolved structures. The final study set comprised apo, DAR_native, DAR_dock, JJB_native, JJB_dock, ROSI, 2YFE, 9L3T, and baicalein. Descriptors unavailable for all systems were analyzed only in the applicable subset.

Hydrogen atoms and standard Amber protonation states were assigned during system preparation. Asp and Glu were modeled as deprotonated, Lys and Arg as protonated, cysteines as neutral reduced Cys, and histidines, including His323 and His449, as neutral HIE tautomers. Baicalein was modeled as a neutral flavone/phenolic ligand state, with phenolic hydroxyl groups retained in protonated form and the chromone carbonyl retained as a neutral acceptor. No alternative protein or ligand-protonation-state sampling was performed in the main production set. The submitted neutral baicalein/HIE histidine model was therefore treated as the production reference condition, and limited ligand-protonation and His323/His449-tautomer sensitivity screens were added as short, non-production replacement controls as described below.

### 2.2 Ligand parameterization and docking route

Receptor preparation and docking used smina (Koes et al., 2013). Ligand parameters used General Amber Force Field 2 (GAFF2) (Wang et al., 2004) with Austin Model 1 bond charge correction (AM1-BCC) partial charges (Jakalian et al., 2002) (AmberTools); missing bonded and non-bonded terms were assigned with parmchk2. Baicalein entered production from a selected smina docking-derived receptor–ligand pose after pose-quality, pocket-localization, steric-compatibility, and ligand-pocket contact inspection. Candidate baicalein poses were not advanced on the docking score alone; the selected-pose was required to remain within the PPARγ ligand-binding pocket, avoid steric clashes with the prepared receptor scaffold, and support chemically plausible polar and hydrophobic contacts. To evaluate whether the absence of persistent canonical AF-2 anchoring was readily attributable to the single selected docking pose used for production, we performed an additional short top-pose sensitivity screen. Three docking poses were tested: the selected docking pose used for production and two alternative high-ranking pocket-localized poses. Each pose was propagated for 30 ns under the same receptor, ligand-protonation, and histidine-tautomer model used for the production baicalein system. The 10- to 30-ns windows were analyzed using the same bound-state criteria and the same strict Ser289/His323/His449/Tyr473 AF-2 anchor hydrogen-bond geometry. This screen was used as a starting-pose sensitivity analysis for the negative AF-2 anchor signal and was not treated as a replacement for the three-replica 100-ns production ensemble.

### 2.3 Topology assembly and solvation

Each receptor–ligand assembly was built with ff14SB (Maier et al., 2015) for the protein and GAFF2 for ligand atoms, solvated in three-site transferable intermolecular potential (TIP3P) water (Jorgensen et al., 1983) water with 10.0 Å padding, and neutralized with counterions; no additional bulk salt was imposed. Box geometry followed the route-specific Amber preparation output. The baicalein production system required two Na^+^ ions, and the representative replica 1 box contained 11,544 TIP3P waters. Amber-format topologies were converted to GROMACS-compatible files with ParmEd, preserving standard Amber non-bonded scaling.

### 2.4 Staged equilibration

Equilibration was performed in OpenMM 8 (Eastman et al., 2024) under constant-number, constant-pressure, constant-temperature (isothermal-isobaric) ensemble (NPT) conditions at 300 K and 1 bar. After energy minimization, each system was relaxed through four preproduction stages: 0.10 ns with strong restraints, 0.15 ns with moderate restraints, 0.15 ns with weak restraints, and 0.20 ns after restraint release. A Langevin integrator was used (friction 2.0 ps^−1^, 2-fs time step), with a Monte Carlo barostat applied every 50 steps. Particle mesh Ewald (PME) electrostatics (Darden et al., 1993) used a 1.2-nm real-space cutoff and an Ewald tolerance of 10^–4^.

Positional restraints were applied to protein Cα atoms and ligand-proximal pocket-shell heavy atoms within 0.6 nm of the ligand at force constants of 2.0, 1.0, and 0.5 kcal mol^−1^ Å^−2^ for the strong, moderate, and weak stages, respectively. Ligand heavy atoms were restrained with an root-mean-square deviation (RMSD) collective variable at 5.0, 2.0, and 1.0 kcal mol^−1^ Å^−2^. A flat-bottom ligand-pocket center-of-mass (COM) restraint used the same sequence with r_0_ = 0.35 nm. All restraints were set to zero during the final 0.20 ns release stage before the export of coordinates for production dynamics.

### 2.5 Unrestrained production dynamics

Production trajectories were propagated in GROMACS (Van Der Spoel et al., 2005) under constant-number, constant-pressure, constant-temperature (isothermalisobaric) ensemble (NPT) at 300 K and 1 bar with a velocity-rescale thermostat, a Parrinello–Rahman barostat, and a 2-fs time step. PME electrostatics used a 1.2-nm real-space cutoff. Bonds involving hydrogen atoms were constrained with the linear constraint solver (Hess, 2008). Three independent 100-ns replicas were generated for each system from distinct velocity seeds. Coordinates were saved every 10 ps, yielding 10,000 frames per replica before window curation.

### 2.6 Trajectory postprocessing and bound-state verification

Periodic-image artifacts were removed by sequential whole-molecule reconstruction, cluster-based repacking, and centering. Processed trajectories were fitted to the protein backbone by least-squares alignment. The 10-90 ns interval was used as the parent production analysis window for structural and dynamical analyses.

For each ligand-bound system, a residue-expanded static pocket heavy-atom set was defined from the first fitted frame by selecting protein residues with at least one heavy-atom within 0.5 nm of the ligand non-hydrogen atom set and retaining the heavy atoms from those residues. Ligand-retention metrics were then computed against this fixed pocket set using three retention criteria: ligand-pocket minimum heavy-atom distance≤0.35 nm, ligand-pocket heavy-atom contact-count ≥ 5 (0.35 nm contact cutoff), and ligand-pocket COM separation ≤3.0 nm. Ligand heavy-atom RMSD ≤ 0.50 nm was monitored as a pose-orientation diagnostic in the QC panel and was not used to define bound-state membership. The corresponding ligand-retention quality-control panel (QC) is summarized in Supplementary Figure S1 and Supplementary Table S1. Global protein-level QC summaries, including protein backbone RMSD, radius of gyration, and solvent-accessible surface area, are provided in Supplementary Figure S2. This separation between retention criteria and pose-orientation diagnostics was important for the baicalein analysis because the starting geometry was docking-derived. Preservation of the initial docked pose was not required for bound-state membership, whereas persistent satisfaction of the distance, contact-count, and COM criteria was required before ligand-dependent descriptors were interpreted.

Frames failing any retention criterion were classified as transient excursion frames and excluded from ligand-dependent downstream analyses when a bound-state-filtered trajectory was required. MM/GBSA calculations used the energy-analysis window defined by the endpoint-energy analysis. An auxiliary ligand-pocket COM trace was retained for diagnostic inspection.

### 2.7 Residue numbering, H12 secondary-structure, and local H12 order

Canonical PPARγ residue numbering was used for all manuscript-level residue labels. For the baicalein production topology, the construct-specific residue offset was 217 (Protein Data Bank (PDB) residue number = simulation residue number + 217). Tyr473 corresponded to simulation residue 256, and the H12 PDB range 465–474 corresponded to simulation residues 248–257, respectively. This mapping was used for trajectory selections, index construction, descriptor extraction, and final residue labels.

The primary H12 order metric was the Define Secondary Structure of Proteins (DSSP) α-helical content from H-state assignments across the mapped H12 segment (Gorelov et al., 2024). Additional local H12 order metrics included DSSP H/G/I helix-like content, backbone i to i + 4 hydrogen-bond persistence, H12 backbone root-mean-square fluctuation (RMSF), and an H12 axial order parameter from the principal axis of H12 Cα coordinates. These H12 order metrics are summarized in Supplementary Figure S3.

### 2.8 AF-2 anchor interaction analysis

Direct protein–ligand hydrogen bonds with the canonical AF-2 anchor residues Ser289, His323, His449, and Tyr473 were monitored over the structural-analysis window. A hydrogen-bond was scored when the donor–acceptor distance was ≤0.35 nm and the D–H–A angle was ≥150°, with periodic-boundary correction. Per-residue occupancy was the fraction of analyzed frames meeting these criteria. A composite AF-2 anchor-panel union score recorded the fraction of frames containing at least one direct ligand hydrogen-bond with any of the four anchor residues. Direct Tyr473–ligand hydrogen bonding and Tyr473–water–ligand bridging were evaluated separately; a bridge-positive frame required one water molecule to satisfy hydrogen-bond geometry with both the Tyr473 hydroxyl and a ligand polar atom.

### 2.9 Pocket-H12 dynamical cross-correlation

Cα-based dynamic cross-correlation matrices were computed over the analysis window with MDAnalysis (Michaud-Agrawal et al., 2011) at stride 10. Pocket-H12 coupling was summarized as the mean absolute cross-correlation, mean(|Cᵢⱼ|), across all pocket-H12 residue pairs. A common mapped pocket-residue set was used for comparability between ligand-bound and apo systems.

### 2.10 MM/GBSA subpocket decomposition and convergence assessment

Trajectory-averaged MM/GBSA calculations (Hou et al., 2011) used gmx_MMPBSA (Valdés-Tresanco et al., 2021) with the GB model (igb = 5) over the energy-analysis window. Pocket residues were partitioned by canonical PPARγ numbering according to the endpoint-energy analysis: Arm I (His323, Tyr327, His449, Leu469, and Tyr473), Arm II (Arg288, Ile341, Ser342, Glu343, Met348, and Leu353), and Arm III (Ile281, Gly284, Cys285, Glu295, and Met364). For each arm, favorable negative per-residue ΔG contributions were summed as stabilizing magnitudes and normalized across Arms I–III to yield arm-level stabilizing fractions; these are MM/GBSA-derived energetic descriptors and are not geometric contact occupancies. Endpoint energies were interpreted as comparative mechanistic descriptors, not absolute cross-system binding-affinity rankings. Convergence was assessed per replica using block consistency, effective sample size, confidence-interval width, lag-1 autocorrelation, moving-block-bootstrap intervals, and trimmed-mean stability. Replica-resolved convergence diagnostics are summarized in Supplementary Figure S4 and Supplementary Table S2; full replica-resolved arm decomposition. Residues outside this Arm I/II/III partition may appear in residue-level ProLIF fingerprint analyses (Section 2.13) but are not assigned to an arm in the MM/GBSA decomposition unless they belong to the predefined canonical arm sets.

### 2.11 Multireference conformational atlas

Six descriptors were extracted per replica over the structural-analysis window: H12 α-helical content, pocket-H12 coupling, β-region E + B content, Ser289 hydrogen-bond occupancy, Tyr473 hydrogen-bond occupancy, and AF-2 anchor-panel union occupancy. Principal component analysis (PCA) axes were fitted on ligand-bound systems only. Apo was excluded from axis fitting and projected afterward using controlled imputation for ligand-dependent descriptors; imputed apo values were used only to obtain apo coordinates and were not interpreted as physical ligand interactions. Arms II and III MM/GBSA stabilizing fractions were retained as mechanistic annotations but not included in the atlas feature set since they are not equivalently defined for apo and ligand-bound systems.

### 2.12 Local Tyr473 free energy profile by umbrella sampling

Umbrella sampling (Ngo and Pham, 2022) was performed along a local Tyr473-engagement coordinate for available reference systems and baicalein. The collective variable was the distance between the Tyr473 hydroxyl oxygen and a ligand hydrogen-bond acceptor selected from the prepared topology of each system. Pull groups contained one atom each. Weighted histogram analysis method (WHAM)-derived PMFs were shifted to each system-specific minimum and reported in kJ mol^−1^. Histogram overlap and bootstrap uncertainty were inspected from the WHAM outputs and are reported in Supplementary Figure S6. These PMFs were interpreted as local Tyr473-engagement profiles, not global ligand-binding free energies. A preliminary amorfrutin 1 Protein Data Bank entry (2YFE) pulling test along the same coordinate did not converge within this protocol; the Supplementary Table S3 2YFE entry therefore reports only the local minimum and endpoint cost for descriptive comparison.

### 2.13 ProLIF interaction fingerprint analysis

Protein–ligand interaction fingerprints were computed independently for each baicalein replica with ProLIF (Bouysset and Fiorucci, 2021) using bound-state-filtered GROMACS trajectories loaded through MDAnalysis. The ProLIF analysis used the common 10- to 90-ns interval after applying the ligand-retention criteria, and frames were sampled at stride 10. Framewise fingerprints were converted into tabular form and collapsed to residue-level occupancies using a per-frame logical OR rule across ligand-residue columns. Interaction labels (HB-A, Hydro, and VdW) denote ProLIF interaction classes after residue-level aggregation and are not interpreted as unique donor or acceptor direction assignments unless explicitly stated. Reported mean occupancies in Section 3 were computed as the arithmetic mean across-replica of replica-level occupancies; replica-level values are shown as integer percentages in Supplementary Figure S5A and as small-decimal-precision values in the processed numerical table. Supplementary Figure S5B ranks interactions using the same consensus interaction summary applied to the replica-level fingerprint table, whereas Section 3 reports arithmetic across-replica means with higher decimal precision; both summaries are derived from the same per replica fingerprint table. Residue labels were converted from simulation numbering to canonical PPARγ numbering using the system-specific offset of 217 during the final ProLIF export.

### 2.14 Replica-level reproducibility analysis

The three baicalein replicas were projected into the multireference atlas and compared with apo-like, partial/non-AF-2, and full-like reference groupings. Per-feature z-score profiles were used to identify descriptors responsible for separation from reference classes. Replica-level Arms II and III MM/GBSA stabilizing fractions were interpreted alongside atlas features but were not equated with kinetics-derived contact descriptors used in state annotation.

### 2.15 Exploratory state partitioning

Exploratory state partitioning (Husic and Pande, 2018) was performed for the eight ligand-bound systems. System-specific feature blocks, lag times, retained dimensions, and convergence diagnostics are shown in Supplementary Table S4. For each system, frames from three replicas were embedded in the selected coordinate space and clustered into 40 microstates with MiniBatchKMeans. Lagged transition counts were accumulated within replicas only and regularized with a small pseudocount; the microstate model was coarse-grained into three macrostates. Macrostate populations were reported as frame occupancies rather than stationary populations because this is the more conservative summary for exploratory models. Chapman–Kolmogorov diagnostics compared predicted and empirical self-transition probabilities at k = 2, 3, 4 lag multiples and were interpreted as model-quality summaries, not a validation of long-timescale kinetics. For baicalein, the selected descriptor set was the extended semantic descriptor set, with a lag time of 0.10 ns and three retained dimensions.

### 2.16 State-resolved descriptor annotation

Per-frame macrostate assignments were integrated with time-matched framewise descriptors describing ligand-pocket geometry, AF-2 hydration, regional contacts, and interaction fingerprints. Macrostate means were computed for a focused descriptor panel: Tyr473 separation; AF-2 direct contact; AF-2 hydrogen-bond count; Arms II and III contact fractions; H3, β, and Ω contacts; and the Ω-flanking/phosphosite-side proxy. The geometric contact-region definitions used for state-level annotation were defined separately from the formal MM/GBSA arm partition and were specified by canonical PDB residue numbers: Arm I included PDB residues 289, 323, 325, 327, 329, 449, 469, and 473; Arm II included PDB residues 281, 284, 285, 288, 326, 330, 333, 339, 341, 342, 348, and 353; and Arm III included PDB residues 280, 295, 340, and 364. Arms II and III contact fractions used in state-resolved annotation were therefore geometric contact-region descriptors from the ligand-pocket dynamic descriptor set and were interpreted separately from the formal MM/GBSA arm-level stabilizing fractions defined in Section 2.10; unless explicitly stated, state-level Arm II/Arm III contact fractions should not be equated with MM/GBSA Arm II/Arm III stabilizing fractions. Because the Ser273-containing segment was unresolved and not rebuilt, Ω-flanking and phosphosite-side descriptors were interpreted as structural proxies adjacent to that region, not as direct measurements of the unresolved Ser273-containing segment.

Each macrostate was evaluated for descriptor-support before mechanistic annotation. A macrostate was included only when at least 10 time-matched descriptor rows remained after merging and the descriptor-assignment match fraction was at least 0.05. For baicalein, all three macrostates passed the interpretability criterion, but the match fractions were low and therefore required conservative interpretation. State A retained 45/300 assignment rows after merging (match fraction 0.150), state B retained 117/1,302 rows (match fraction 0.0899), and state C retained 103/801 rows (match fraction 0.1286). All three states were retained in the descriptor-level summary, with state-specific interpretation bounded by this descriptor-support assessment.

### 2.17 Ligand-protonation and His323/His449-tautomer sensitivity screening

To evaluate whether the AF-2 anchor-negative result was immediately dependent on the selected neutral ligand and HIE323/HIE449 histidine model, short chemistry-state sensitivity screens were performed. Two review-critical chemistry states were tested: 7-O monoanionic baicalein with HIE323/HIE449 and neutral baicalein with HID323/HID449. The 7-O monoanion was generated from the mapped 7-O phenolic group; atom-level preparation details are provided in the Supplementary Material. Each chemistry-state was propagated in three 50-ns screening replicas and analyzed over 10–50 ns using the same ligand-retention criteria and AF-2 hydrogen-bond geometry used for the production descriptor analysis. These simulations were used as chemistry-state sensitivity screens and were not treated as full production replacements.

### 2.18 Computational tools and AI use disclosure

Production simulations used GROMACS (Van Der Spoel et al., 2005). Preproduction equilibration used OpenMM 8 (Eastman et al., 2024). Ligand parameterization used AmberTools with GAFF2 (Wang et al., 2004) and AM1-BCC charges (Jakalian et al., 2002). Amber-to-GROMACS topology conversion used ParmEd. Docked controls and target docking-derived poses used smina (Koes et al., 2013). Structural and energetic analyses used GROMACS utilities, MDAnalysis (Michaud-Agrawal et al., 2011), gmx_MMPBSA (Valdés-Tresanco et al., 2021), ProLIF (Bouysset and Fiorucci, 2021), NumPy (Harris et al., 2020), pandas (McKinney, 2010), scikit-learn (Pedregosa et al., 2011), and Matplotlib (Hunter, 2007). Visualization used PyMOL (Seeliger and de Groot, 2010). During manuscript preparation, AI-assisted tools were used for language polishing of the human-written manuscript text. All scientific content, data interpretation, simulation analysis, conclusions, and final manuscript text were reviewed and approved by the authors, who take full responsibility for the integrity and accuracy of the work.

## 3 Results

### 3.1 Baicalein showed pocket retention with replica-dependent contact-criterion excursions and pose heterogeneity

Baicalein was analyzed in three independent 100-ns production replicas over the common 10–90 ns analysis window. Canonical PPARγ residue numbering was used throughout Section 3. Across the three replicas, baicalein satisfied the minimum-distance and COM components of the ligand-retention criteria for most analyzed frames, whereas the contact-count criterion revealed replica-dependent pocket-shell contact excursions. Retained frame fractions were 94.20%, 94.00%, and 71.64% in replicas 1–3, respectively, with excursion fractions of 5.80%, 6.00%, and 28.36%, respectively (Supplementary Figure S1; Supplementary Table S1). The ligand-pocket minimum-distance averaged 0.312 ± 0.003 nm across-replica means, with individual means of 0.309 ± 0.012, 0.309 ± 0.012, and 0.311 ± 0.013 nm in retained frames. The overall observed minimum-distance range in the analysis window was 0.256–0.357 nm; only replica 3 showed a small number of minimum-distance criterion failures, whereas most excursions were assigned by the contact-count criterion.

Ligand-pocket contact counts were the principal limiting retention metric. Retained frame contact means were 10.06 ± 3.42, 10.68 ± 3.47, and 8.07 ± 2.73 contacts for replicas 1 to 3, respectively, while contact-criterion failures accounted for 464, 480, and 2,269 frames in the 10–90 ns analysis window. The true ligand-pocket COM distance remained well below the 3.0 nm criterion, with retained frame means of 0.16 ± 0.08, 0.30 ± 0.04, and 0.49 ± 0.21 nm. Ligand heavy-atom RMSD was higher in replica 3 than in replica 1 and replica 2, giving analysis window means of 0.196 ± 0.126, 0.209 ± 0.034, and 0.827 ± 0.220 nm and an across-replica mean of 0.410 ± 0.361 nm (Supplementary Figure S1 and Supplementary Table S1). Because RMSD was used as a pose diagnostic and not as a retention criterion, the elevated replica 3 RMSD was interpreted together with the contact-criterion excursions as in-pocket pose reorganization and reduced pocket-shell contact persistence rather than COM-defined ligand release. Ligand-dependent downstream descriptors should therefore be interpreted on the corresponding bound-state-filtered frame set.

### 3.2 The reference atlas summarized baicalein as AF-2-anchor silent under the selected docking-derived model

**FIGURE 1:**
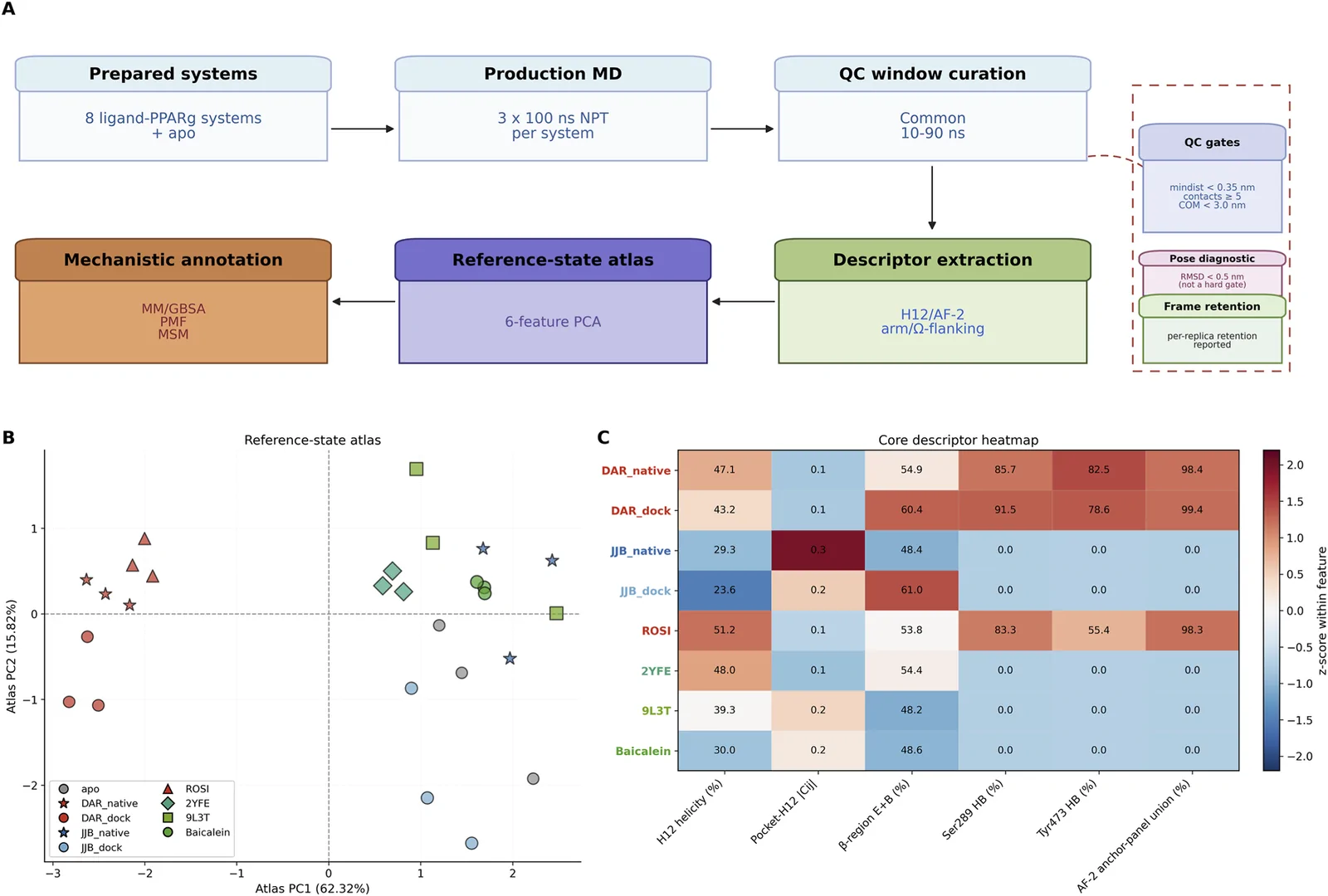
Multireference conformational descriptor analysis of baicalein and peroxisome proliferator-activated receptor γ (PPARγ) comparator systems. **(A)** Summary of the simulation design, common 10–90 ns analysis window, ligand-retention filtering, reference-state descriptor projection, and downstream structural interpretation. **(B)** The replica-level PCA fitted on ligand-bound systems using six atlas descriptors, with apo projected passively after controlled imputation of ligand-dependent features. **(C)** System-level descriptor means. Baicalein projected away from full-agonist references and showed a measurable helix 12 (H12) order with 0.00% Ser289, Tyr473, and activation function-2 (AF-2) anchor-panel union hydrogen-bond occupancy. Because anchor hydrogen-bond variables are included in this atlas, the PCA is interpreted as a visual descriptor summary; a control PCA excluding these anchor variables is provided as a robustness analysis.

In the multireference descriptor analysis, the three baicalein replicas projected away from the full-agonist cluster. The overall analysis workflow is summarized in Figure 1A. Their PC1 values were 1.690, 1.696, and 1.610, and their PC2 values were 0.310, 0.242, and 0.374 (Figure 1B). At the system level, baicalein showed 30.01% H12 alpha helicity, pocket-H12 |Cij| of 0.170, and beta-region E + B content of 48.57% (Figure 1C). In contrast to the full-like reference systems, the Ser289 HB, Tyr473 HB, and AF-2 anchor-panel union occupancies were all 0.00%. Because these anchor variables are part of the atlas feature set, the PCA was interpreted as a descriptor summary rather than as an independent discovery of AF-2 silence.

The anchor-panel descriptors separated baicalein from the full-agonist references. DAR_native, DAR_dock, and ROSI showed AF-2 union occupancies of 98.42%, 99.42%, and 98.33%, respectively, whereas baicalein, JJB_dock, 2YFE, and 9L3T were at 0.00%, and JJB_native was near zero at 0.04% (Figure 1C). A control PCA excluding AF-2 anchor variables, including Ser289 HB, Tyr473 HB, and AF-2 anchor-union features, was generated as a robustness control. In that analysis, the excluded anchor variables were not used to define PC1 or PC2; baicalein remained evaluable using H12 helicity, pocket-H12 coupling, beta-region E + B content, and arm-level MM/GBSA descriptor fractions. The anchor-free analysis is reported as a robustness control rather than as a new mechanistic claim. Additional bootstrap and leave-one-replica robustness analyses of atlas placement and descriptor classification are provided in Supplementary Figure S8.

### 3.3 Baicalein lacked canonical AF-2 anchor hydrogen bonds but retained local H12 order

**FIGURE 2:**
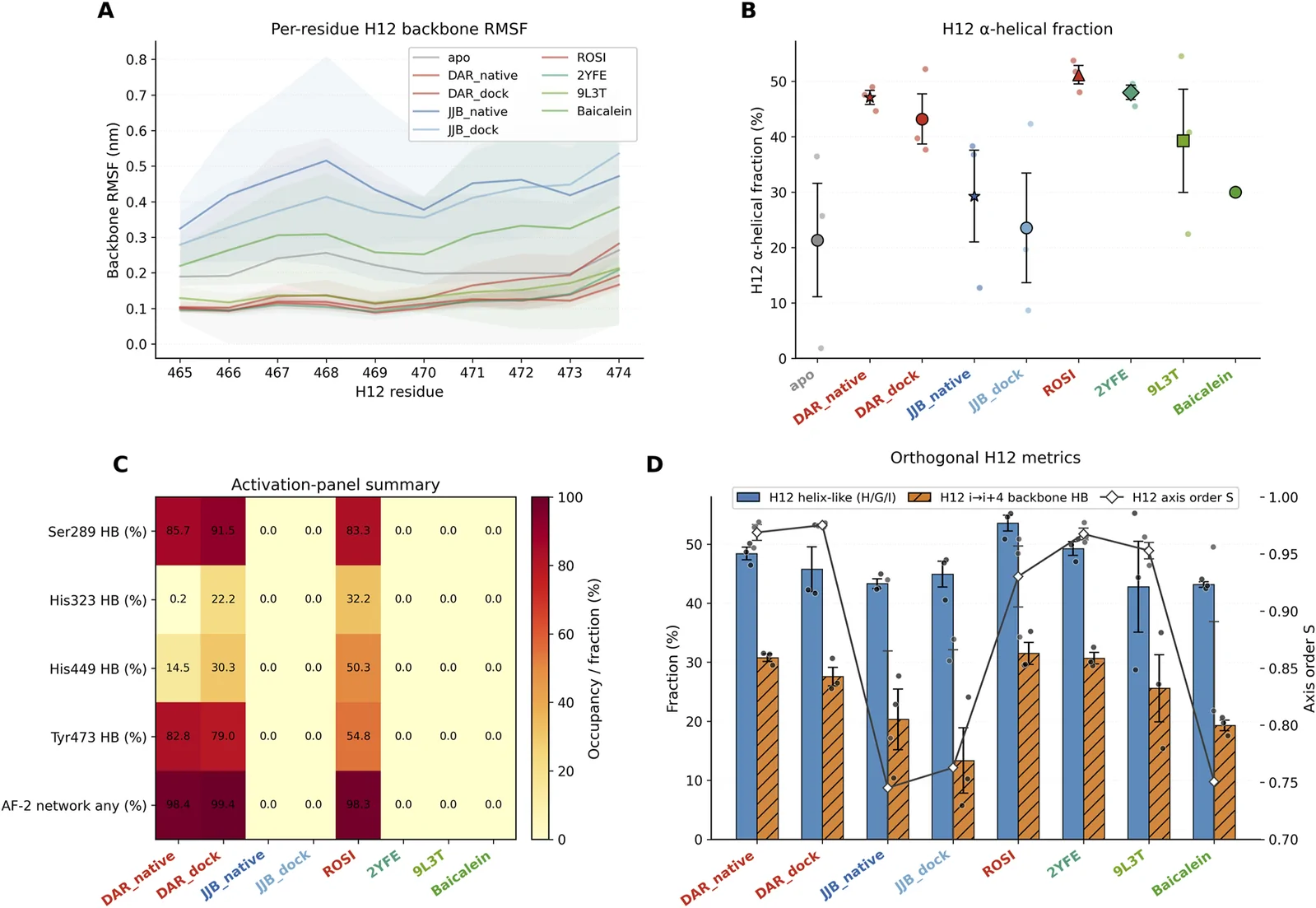
Helix 12 (H12) local-order and activation function-2 (AF-2) anchor descriptors. **(A)** Per-residue H12 backbone root-mean-square fluctuation (RMSF) across comparator systems. **(B)** Replica-level and system-level H12 α-helical fractions. **(C)** System-level hydrogen-bond occupancies for Ser289, His323, His449, Tyr473, and the AF-2 anchor-panel union. **(D)** Orthogonal H12 order metrics, including H/G/I helix-like fraction, i→i+4 backbone hydrogen-bond persistence, and H12 axis order parameter S. Baicalein retained measurable local H12 order, whereas direct hydrogen bonds to Ser289, His323, His449, Tyr473, and the AF-2 anchor-panel union remained at 0.00%.

Direct hydrogen bonds between baicalein and the canonical AF-2 panel residues were absent under the geometric criteria used in this analysis. Per-residue H12 backbone fluctuations across comparator systems are shown in Figure 2A. The mean occupancies for Ser289 HB, His323 HB, His449 HB, Tyr473 HB, and the AF-2 network union were all 0.00% across the analyzed replicas (Figure 2C). This zero signal was retained despite local H12 organization.

Replica-level H12 α-helicity values were 30.08%, 29.61%, and 30.35%, yielding a system mean of 30.01% (Figure 2B). Orthogonal H12 descriptors gave 43.17% H/G/I helix-like content, 19.29% H12 i → i + 4 backbone hydrogen-bond persistence, and an H12 axial order parameter of 0.751 (Figure 2D). These values support local H12 ordering while preserving the distinction that baicalein did not form the direct AF-2 anchor hydrogen-bond network detected for the full-agonist references.

### 3.4 Arm-resolved decomposition and ProLIF fingerprints defined the baicalein recognition pattern

**FIGURE 3:**
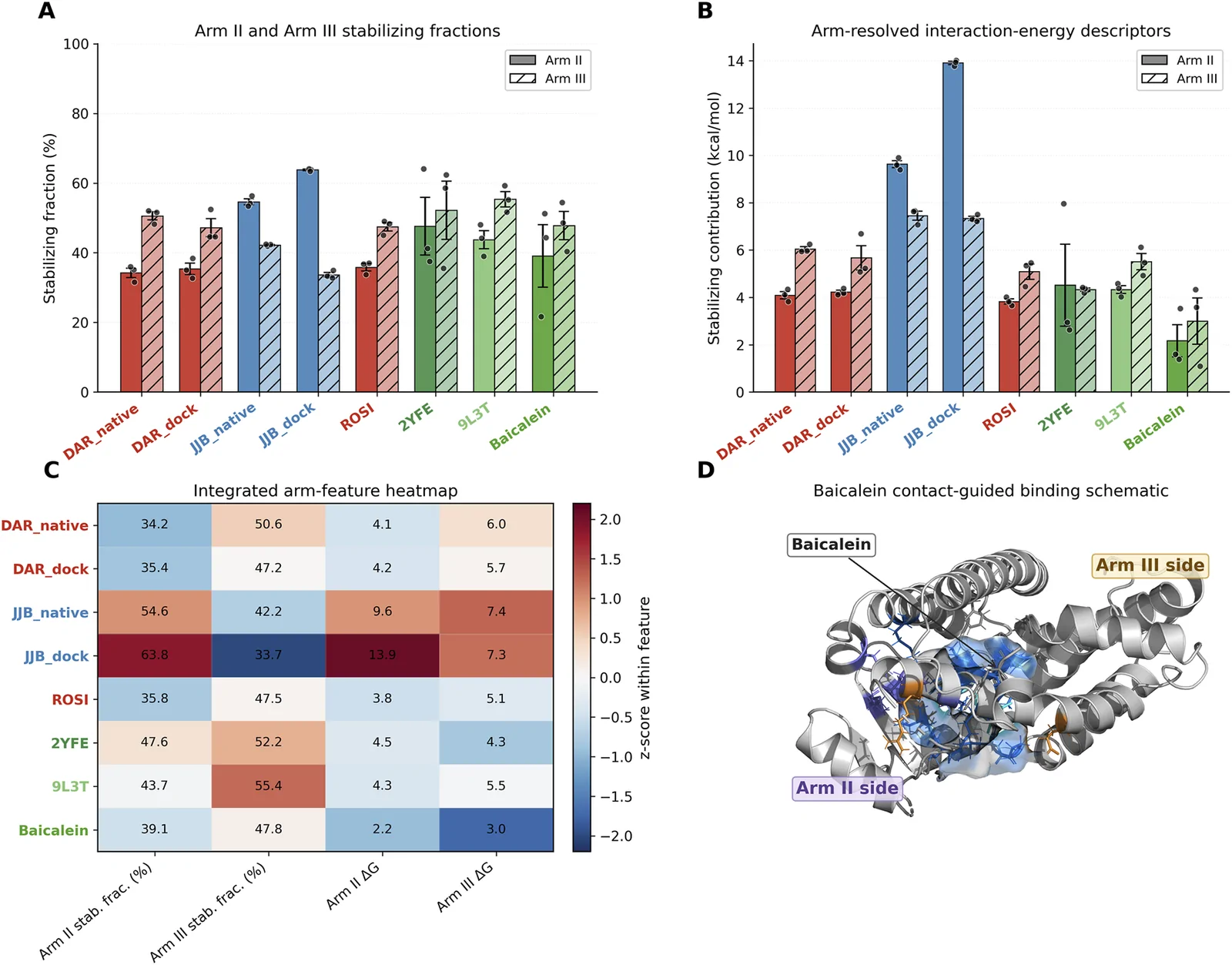
Arm-resolved molecular mechanics/generalized Born surface area (MM/GBSA) descriptors and representative baicalein binding pose. **(A–C)** Comparison of Arms I, II, and III stabilizing fractions; arm-level stabilizing contributions; and an integrated arm-feature heat map. Baicalein showed mixed Arm II/Arm III stabilizing participation, with Arms I, II, and III contributing 13.10%, 39.10%, and 47.80% across three replicas, respectively. **(D)** The representative pose image with canonical peroxisome proliferator-activated receptor γ (PPARγ) residue labels checked against the resolved offset mapping.

Arm-resolved MM/GBSA decomposition showed a mixed Arms II and III stabilizing profile for baicalein. Across replicas, Arms I, II, and III accounted for 13.10% ± 14.86%, 39.10% ± 15.49%, and 47.80% ± 7.03% of stabilizing contributions, respectively (Figure 3A–C and Supplementary Table S5). The corresponding stabilizing energy magnitudes were 2.17 kcal mol^−1^ for Arm II and 3.00 kcal mol^−1^ for Arm III (Figure 3B). Replica-level decomposition retained this mixed profile, with Arm II contributing 51.28%, 21.66%, and 44.36% in replica 1 to replica 3, and Arm III contributing 40.41%, 48.58%, and 54.42%, respectively (Supplementary Table S5). The representative baicalein binding pose and the Arm II/Arm III sides are shown in Figure 3D.

**FIGURE 4:**
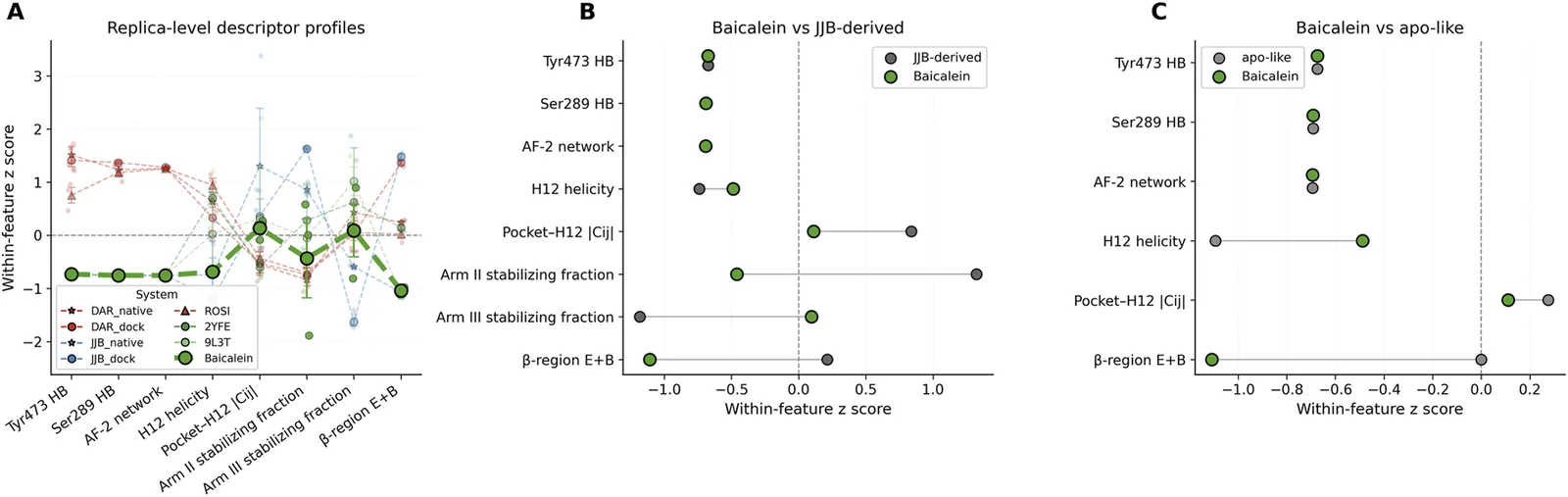
Replica-level reproducibility of the baicalein descriptor profile. **(A)** Replica-level within-feature z-score profiles across the atlas-plus-arm descriptor panel. **(B)** Paired within-feature z-score comparison between baicalein and the JJB-derived reference group. **(C)** Paired within-feature z-score comparison between baicalein and the apo-like reference. Leave-one-replica sensitivity analysis showed that the zero activation function-2 (AF-2) anchor signal, helix 12 (H12) helicity, pocket-H12 coupling, and β-region E+B content were insensitive to exclusion of replica 3, whereas contact retention and the normalized Arm I molecular mechanics/generalized Born surface area (MM/GBSA) descriptor were more replica-sensitive.

Total MM/GBSA replica means were −30.77, −28.44, and −32.92 kcal mol^−1^ for replica 1 to replica 3, with an across-replica mean of −30.71 ± 2.24 kcal mol^−1^ (Supplementary Figure S4 and Supplementary Table S2). The per replica convergence labels were strong for replica 1 arm rows and fair for replica 2 and replica 3 arm rows in Supplementary Table S5. These endpoint-energy values were used as convergence-aware descriptors rather than absolute binding-affinity estimates.

ProLIF fingerprints supported the same residue-level recognition pattern in the bound-state-filtered frame set (Supplementary Figure S5). The highest mean baicalein interaction occupancies were CYS285 (VdW) at 59.16% with replica values of 39%, 62%, and 76%; ILE341 (VdW) at 44.56% with 1%, 52%, and 81%; LEU330 (VdW) at 39.22% with 34%, 59%, and 25%; LEU333 (VdW) at 37.88% with 39%, 44%, and 31%; ILE326 (VdW) at 36.12% with 58%, 50%, and 1%; and MET329 (VdW) at 33.93% with 59%, 34%, and 9%. ARG288 (VdW) and SER289 (VdW) were also detected at 32.50% and 27.69%, respectively. These ProLIF occupancies describe residue-level interaction classes after aggregation and are distinct from the direct hydrogen-bond geometry used for the AF-2 anchor analysis. Therefore, the Ser289 VdW contact signal does not alter the zero Ser289 AF-2 hydrogen-bond result. Replica-level descriptor profiles and comparisons with JJB-derived and apo-like systems are summarized in Figures 4A–C. Leave-one-replica sensitivity analysis showed that the zero AF-2 anchor signal, H12 helicity, pocket-H12 coupling, and β-region E + B content were insensitive to exclusion of the third replica, whereas contact retention and the normalized Arm I MM/GBSA descriptor were more replica sensitive (Supplementary Figure S9).

### 3.5 The local Tyr473 engagement profile showed a shifted baicalein minimum

**FIGURE 5:**
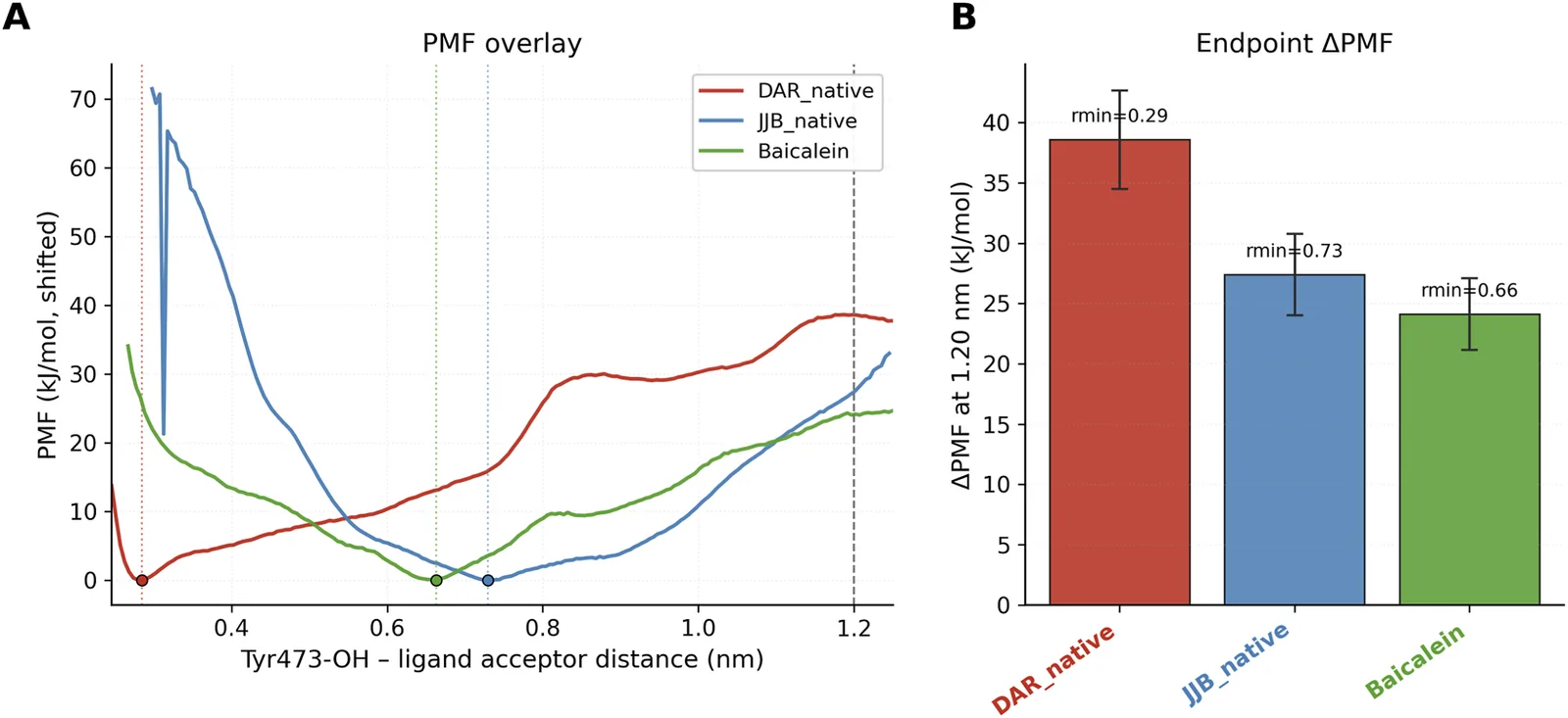
Local Tyr473-engagement potential-of-mean-force (PMF) profiles. **(A)** Overlay of PMF profiles along the Tyr473 hydroxyl-to-ligand-acceptor coordinate after shifting each profile to its system-specific minimum. **(B)** Endpoint ΔPMF at 1.20 nm for DAR_native, JJB_native, and baicalein; labels above bars indicate the system-specific PMF-minimum distance (rmin). Baicalein showed a local minimum at 0.66 nm and a ΔPMF at 1.20 nm of 24.18 kJ mol−1, consistent with a displaced Tyr473 geometry relative to the near-contact full-agonist reference. This coordinate is a local Tyr473-engagement descriptor, not a global binding free-energy estimate.

The Tyr473 OH to ligand acceptor PMF coordinate placed baicalein away from the near-contact full-agonist geometry. DAR_native had a PMF minimum at 0.29 nm and ΔPMF at 1.20 nm of 38.62 kJ mol^−1^, whereas baicalein had a shifted minimum at 0.66 nm and ΔPMF at 1.20 nm of 24.18 kJ mol^−1^ (Figure 5 and Supplementary Table S3). JJB_native, used as the partial-like comparator in Figure 5, had a minimum at 0.73 nm and ΔPMF at 1.20 nm of 27.45 kJ mol^−1^. The baicalein PMF profile is therefore consistent with a displaced local Tyr473 geometry relative to the direct Tyr473-centered full-agonist reference and with the absent Tyr473 hydrogen-bond signal in unbiased trajectories. This PMF coordinate was interpreted as a local Tyr473 engagement descriptor rather than a whole ligand unbinding free energy or global affinity ranking (Supplementary Figure S6 and Supplementary Table S3).

### 3.6 Exploratory coarse graining resolved baicalein state heterogeneity without assigning definitive kinetic rates

**FIGURE 6:**
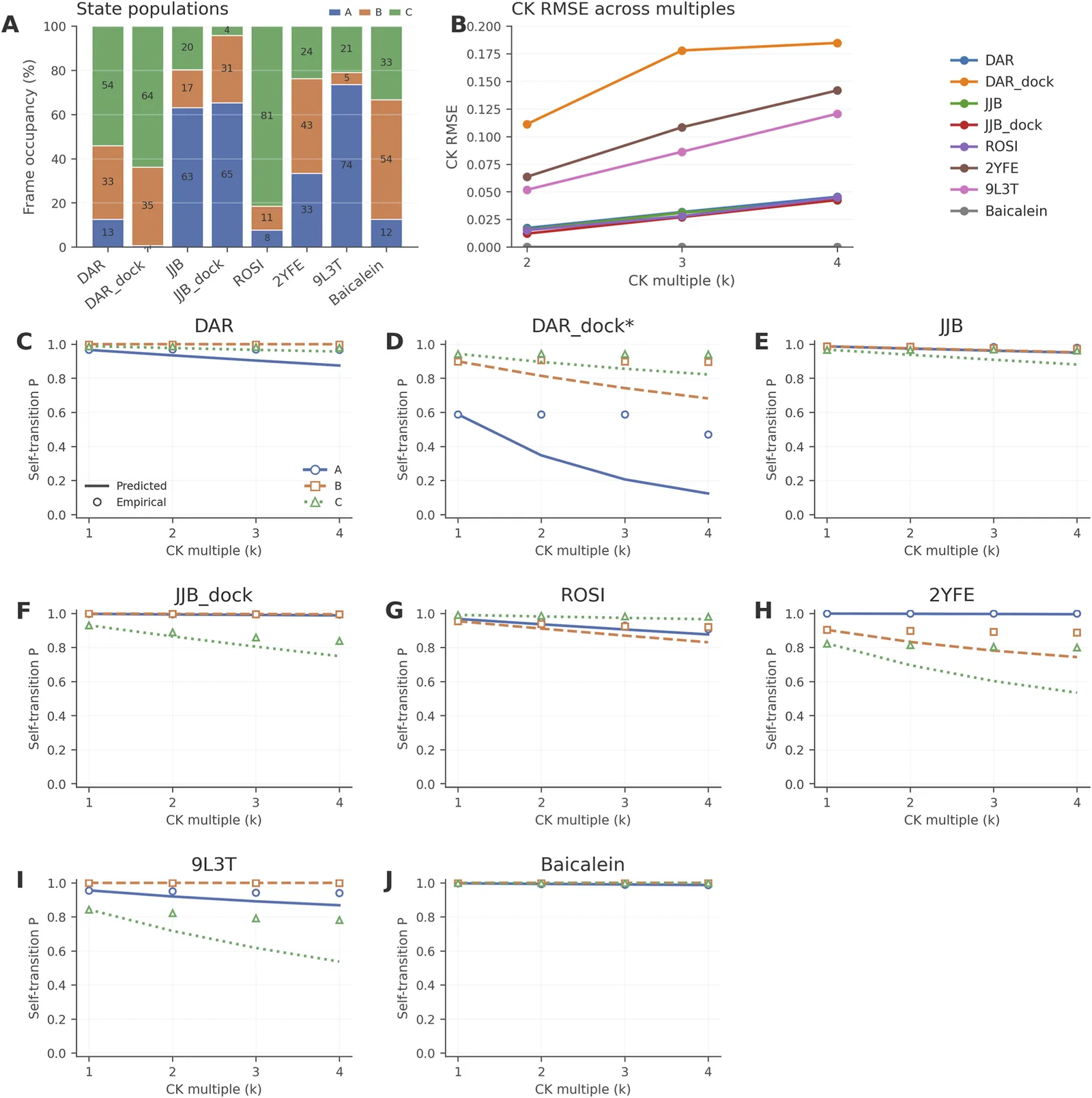
Exploratory coarse-grained state annotation for ligand-bound peroxisome proliferator-activated receptor γ (PPARγ) systems. **(A)** Frame-occupancy state populations for states A-C across the eight systems. **(B)** Chapman-Kolmogorov (CK) root-mean-square error (RMSE) across lag multiples 2-4. **(C-J)** Predicted and empirical state-specific self-transition probabilities across CK lag multiples for DAR_native, DAR_dock, JJB_native, JJB_dock, ROSI, 2YFE, 9L3T, and baicalein, respectively. State populations are reported as frame occupancies rather than stationary thermodynamic populations. For baicalein, the model used the extended descriptor set, a lag time of 0.10 ns, three retained dimensions, 40 microstates, and three macrostates, with descriptor-matched frame occupancies of 12.48%, 54.18%, and 33.33% for states A-C. Supplementary Table S4 reports the corresponding macrostate-assignment counts.

The exploratory coarse-grained model for baicalein used the extended semantic feature block, a lag time of 0.10 ns, three retained dimensions, 40 microstates, and three macrostates (Figure 6 and Supplementary Table S4). CK RMSE values were 0.00001, 0.00002, and 0.00003 at 2×, 3×, and 4× lag multiples, respectively (Figure 6B). Supplementary Table S4 lists the model recipe and macrostate assignment summary; the reported state populations were treated as frame occupancies rather than stationary thermodynamic populations or definitive kinetic rates.

**FIGURE 7:**
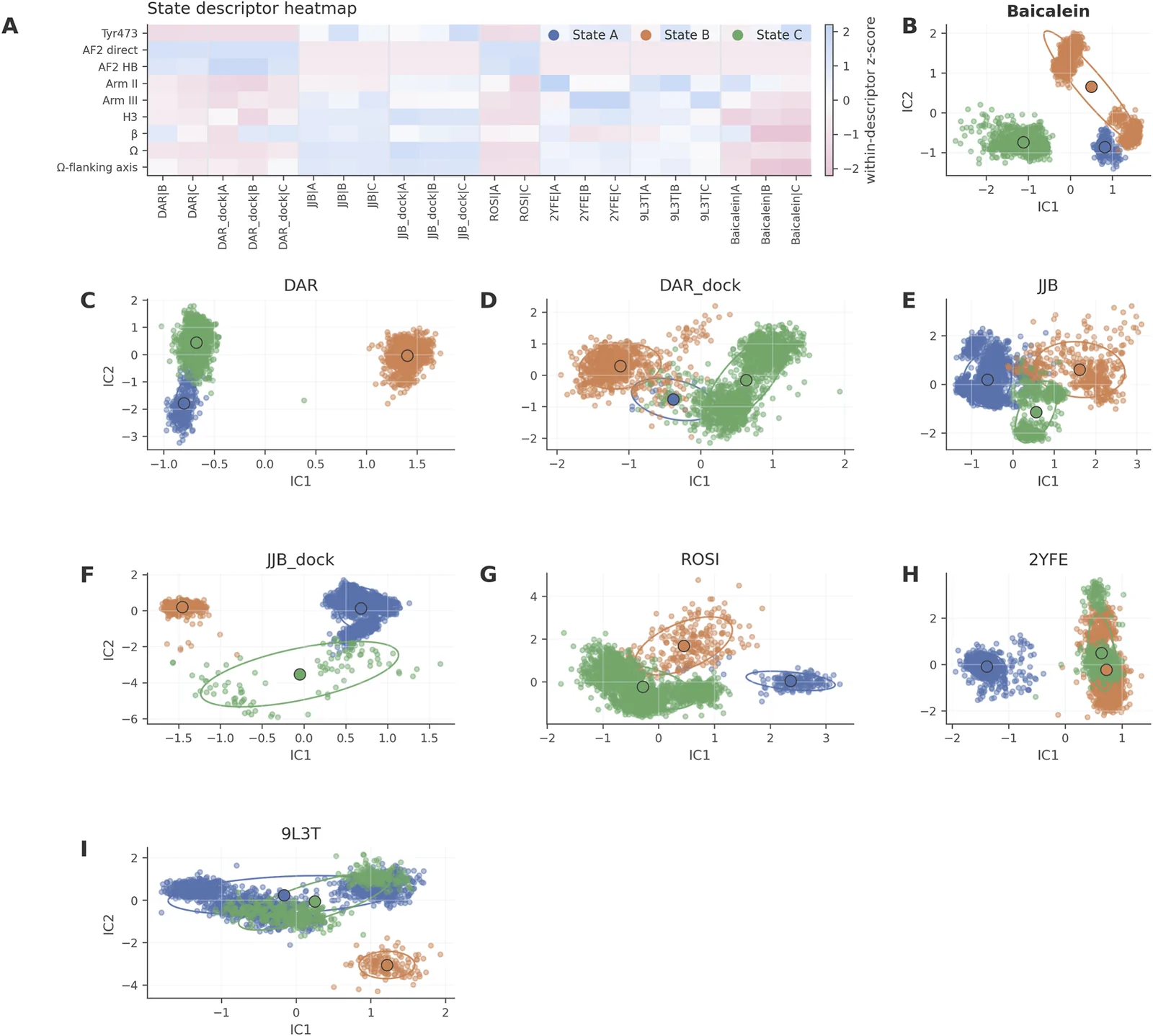
State-resolved descriptor annotation of exploratory macrostates. **(A)** Within-descriptor z-score heat map of descriptor-supported macrostate means across the analyzed systems. **(B-I)** Projected slow-coordinate scatter plots (IC1 and IC2) showing state partitions for baicalein, DAR_native, DAR_dock, JJB_native, JJB_dock, ROSI, 2YFE, and 9L3T, respectively. For baicalein, all three states met the minimum descriptor-support criterion, but descriptor-match fractions were low; therefore, state-level interpretation was restricted to conservative descriptive annotation.

For baicalein, the descriptor-matched state set contained states A, B, and C occupancies of 12.48%, 54.18%, and 33.33%, respectively (Figures 6A and 7). The corresponding model-assignment counts before descriptor matching are provided in Supplementary Table S4 and were used only to document the exploratory state model, not to infer kinetic rates.

### 3.7 Descriptor-supported baicalein state summaries remained anchor-negative despite limited descriptor-matched support

All three baicalein macrostates met the descriptor-support criterion and were therefore retained for descriptor-level annotation (Figure 7 and Supplementary Figure S7). The descriptor merge fractions were 0.150 for state A, 0.0899 for state B, and 0.1286 for state C, with descriptor-supported frame counts of 45, 117, and 103, respectively. These low match fractions are reported in Supplementary Figure S7 and require conservative state-level interpretation.

State-resolved descriptors showed persistent absence of direct AF-2 hydrogen bonding. The AF-2 direct union was 0.00 in all three states, the AF-2 HB count was 0.00 in all three states, and the AF-2 bridge union was 0.00 in all three states (Figure 7A). Tyr473 minimum distances increased from state A to state C, with values of 0.80, 1.33, and 2.22 nm. Ser289 minimum distances were 0.42, 0.62, and 0.36 nm for states A–C, respectively, but these distances did not correspond to direct AF-2 hydrogen-bond occupancy.

The state annotations also resolved changes in arm contact distribution and H12 geometry. Geometric Arm II contact-region fractions remained the dominant arm descriptor in all three states, decreasing from 79.73% in state A to 71.10% in state B and 65.80% in state C. Arm III contact-region fractions were 13.15%, 6.84%, and 5.65%, whereas Arm I increased from 7.11% to 22.06% and 28.55% across the same states (Figure 7). H12 tilt values were 12.27°, 24.09°, and 35.41° for states A–C, respectively, and H12 H3 COM distances were 2.70, 2.15, and 2.87 nm. Thus, the state model resolved baicalein conformational heterogeneity through arm and H12-associated descriptors, whereas the direct AF-2 anchor hydrogen-bond signal remained absent in every descriptor-supported state.

### 3.8 Short top-pose sensitivity screening did not recover persistent canonical AF-2 anchoring

To test whether the absence of persistent canonical AF-2 anchoring was unique to the selected docking pose used for production, we performed short 30-ns screening simulations from three docking poses: the selected docking pose used for production and two alternative high-ranking pocket-localized poses. The 10- to 30-ns windows were analyzed with the same bound-state criteria and strict AF-2 anchor hydrogen-bond geometry used for the production analysis.

All three short simulations remained pocket-associated, with retained fractions of 97.60%, 99.95%, and 99.95% for pose01_selected, pose02_alt_cluster, and pose03_alt_cluster, respectively. No canonical Ser289/His323/His449/Tyr473 anchor hydrogen-bond was detected in any bound-state-filtered screening trajectory, and the AF-2 anchor-union occupancy was 0.00% in all three screens. Tyr473–ligand acceptor distances remained displaced, with mean values of 1.404, 1.502, and 1.327 nm (Supplementary Table S6 and Supplementary Figure S10).

The dominant pocket contacts varied across starting poses, indicating pose-dependent local packing. However, none of the alternative short screens recovered persistent canonical AF-2 anchoring. These results support the interpretation that the negative AF-2 anchor signal is not readily attributable to the single selected docking pose while remaining limited to short screening simulations rather than full production replacements.

### 3.9 Ligand-protonation and His323/His449-tautomer sensitivity did not recover persistent canonical AF-2 anchoring

To test whether the absence of persistent canonical AF-2 anchoring was dependent on the neutral baicalein/HIE323–HIE449 chemistry model, we performed limited 50-ns chemistry-state sensitivity screens. The 7-O monoanionic baicalein/HIE323–HIE449 screens remained pocket-associated, with retained fractions of 100.00%, 99.95%, and 100.00% across three replicas. AF-2 anchor-union occupancies were 0.05%, 0.00%, and 0.00%, respectively, and Tyr473 hydrogen-bond occupancy remained 0.00% in all three replicas. The neutral baicalein/HID323–HID449 screens also remained pocket-associated, with retained fractions of 98.35%, 99.30%, and 89.70% across three replicas. No canonical AF-2 anchor-union event was detected in this histidine-tautomer sensitivity set, and Tyr473 hydrogen-bond occupancy was 0.00% in all replicas. Thus, the tested ligand-protonation and His323/His449-tautomer variants did not recover a persistent canonical Ser289/His323/His449/Tyr473 AF-2 anchor network. These sensitivity screens reduce the likelihood that the negative AF-2 anchor result is a direct artifact of the neutral ligand or HIE histidine model while remaining limited to the tested chemistry states and short screening simulations (Supplementary Table S7 and Supplementary Figure S11).

## 4 Discussion

### 4.1 The selected baicalein model supports pocket association without persistent canonical AF-2 anchoring

The central observation from the selected baicalein simulations is that ligand-pocket association and canonical AF-2 anchoring were separable under the sampled conditions. Baicalein remained spatially associated with the ligand-binding pocket by minimum-distance and COM criteria, but contact-count retention was heterogeneous, especially in replica 3; therefore, the absence of AF-2 anchor hydrogen bonds should be evaluated on the bound-state-filtered frame set rather than simply attributed to ligand release. The zero occupancies for Ser289, His323, His449, Tyr473, and the AF-2 anchor-union describe a selected docking-derived ensemble in which the canonical full-agonist anchor network was not populated (Kroker and Bruning, 2015; Nolte et al., 1998; Bruning et al., 2007; Porskjær Christensen and Bahij El-Houri, 2018).

This distinction constrains the mechanistic interpretation. Baicalein did not behave as a weakened version of the full-agonist anchor pattern within the sampled trajectories. The direct anchor network was absent under the same geometric criteria that identified anchor-positive behavior in the full-agonist reference systems. The observed H12 descriptors therefore need to be interpreted separately from the AF-2 assembly. Baicalein retained local H12 organization, but this local-order was not coupled to direct Ser289, His323, His449, or Tyr473 hydrogen bonding (Kroker and Bruning, 2015; Nolte et al., 1998; Bruning et al., 2007; Porskjær Christensen and Bahij El-Houri, 2018; Hughes et al., 2012; Hughes et al., 2014; Chrisman et al., 2018; Shang et al., 2019; Shang et al., 2020).

The relevant mechanistic distinction is therefore not between an ordered and a collapsed H12 state. It is between local H12 structural persistence and the formation of the coactivator-facing AF-2 anchor network. The baicalein ensemble supports the former but not the latter. This interpretation avoids treating secondary-structure persistence as a direct proxy for the agonist-like AF-2 assembly. It is also consistent with broader PPARγ structural-dynamics literature showing that ligand efficacy, H12 behavior, AF-2 surface organization, alternate-site engagement, and conformational ensembles cannot be reduced to a single binary H12 state (Hughes et al., 2012; Hughes et al., 2014; Chrisman et al., 2018; Shang et al., 2019; Shang et al., 2020).

### 4.2 Baicalein recognition is organized through mixed Arms II and III engagement rather than Tyr473-centered anchoring

The interaction pattern explains how baicalein can remain pocket-associated while lacking persistent canonical AF-2 anchoring. Arm-resolved decomposition and residue-level fingerprints indicate a mixed Arms II and III recognition mode. The dominant ProLIF signals were mainly VdW contacts involving pocket residues such as CYS285, ILE341, LEU330, LEU333, ARG288, and ILE326. These contacts provide a structural basis for pocket association but do not correspond to the direct hydrogen-bond architecture of a full-agonist AF-2 anchor (Nolte et al., 1998; Bruning et al., 2007; Bouysset and Fiorucci, 2021).

The Ser289 contact signal requires the same separation of interaction types. Baicalein showed a Ser289 VdW contact in the residue-level fingerprint, but this does not conflict with the zero Ser289 hydrogen-bond occupancy. The ProLIF fingerprint reports aggregated residue-interaction classes (Bouysset and Fiorucci, 2021), whereas the AF-2 anchor analysis requires direct hydrogen-bond geometry. A non-polar or packing contact near an AF-2-region residue therefore does not convert the ensemble into an anchor-positive state.

The local Tyr473 profile is consistent with this contact framework. Baicalein favored a displaced Tyr473 geometry rather than a short-distance Tyr473-centered anchor. The PMF coordinate was limited to Tyr473 hydroxyl-to-ligand acceptor separation, so it should not be interpreted as a global binding free energy (Ngo and Pham, 2022). Within that local scope, it supports the same conclusion as the unbiased trajectories: Baicalein remains locally pocket-associated after bound-state filtering, whereas Tyr473 is not organized into the canonical agonist anchor geometry.

### 4.3 Baicalein sampled an intermediate locally ordered but anchor-negative reference space

The atlas placement provides a broader context for the baicalein ensemble. Baicalein did not cluster with the full-agonist references that showed high AF-2 anchor occupancy. It instead occupied a non-canonical reference space characterized by absent AF-2 anchor-panel hydrogen bonding, preserved β-region content, modest pocket-H12 coupling, and intermediate local H12 order (Kroker and Bruning, 2015; Bruning et al., 2007; Hughes et al., 2012; Hughes et al., 2014; Chrisman et al., 2018; Shang et al., 2019; Shang et al., 2020).

This combination is mechanistically distinct from simple ligand dissociation or H12 collapse, but it should be interpreted within the selected docking-derived starting-pose and protonation model. Ligand dissociation would weaken the basis for interpreting pocket descriptors, but the minimum-distance and COM components of the retention criteria remained satisfied for most analyzed frames, and ligand-dependent descriptors were evaluated on bound-state-filtered frames. H12 collapse would imply loss of local-order, but the H12 metrics retained measurable organization. A hidden full-agonist-like subpopulation would be expected to produce at least some direct AF-2 anchor signal under the applied criteria, but the anchor-panel occupancies remained zero. The more constrained interpretation is that the selected baicalein model sampled a pocket-associated, locally ordered, anchor-negative ensemble with replica-dependent contact persistence.

This interpretation also clarifies the role of pocket-H12 coupling. The coupling signal was not consistent with a simple full-agonist-like mechanism in which ligand-binding directly locks H12 into a canonical AF-2 assembly. Instead, baicalein appears to preserve local H12 structure without forming the Tyr473-centered and Ser289/His323/His449-associated anchor network (Kroker and Bruning, 2015; Nolte et al., 1998; Bruning et al., 2007; Porskjær Christensen and Bahij El-Houri, 2018; Hughes et al., 2012; Hughes et al., 2014; Chrisman et al., 2018; Shang et al., 2019; Shang et al., 2020).

### 4.4 Sampled heterogeneity remained within a regime without persistent canonical AF-2 anchoring

The replica and state-resolved analyses indicate that baicalein does not occupy a single rigid pose in the selected model. The elevated ligand RMSD in one replica, the contact-criterion excursions, the arm contact redistribution, and the state-resolved differences in Tyr473 separation and H12 geometry support in-pocket heterogeneity. This heterogeneity, however, remained within a regime without persistent canonical AF-2 anchoring in the descriptor-supported and bound-state-filtered frames.

The exploratory state partition did not reveal a descriptor-supported macrostate with canonical AF-2 hydrogen bonding. Instead, the states differed mainly in the distribution of Arms I, II, and III contact-region fractions; Tyr473 separation; and H12-associated geometry. This indicates that the time-averaged AF-2 silence was not masking a sampled full-agonist-like basin within the analyzed trajectories. The sampled variation is better described as redistribution among non-canonical pocket-associated substates.

The state model should still be read conservatively (Husic and Pande, 2018). The macrostate populations are frame occupancies rather than stationary thermodynamic populations, and coarse graining was exploratory. State-level mechanistic interpretation is justified only where descriptor-support was available; for baicalein, all three states passed the minimum interpretability criterion, but the low descriptor merge fractions require cautious reading. Within that boundary, the state analysis supports the same conclusion as the time-averaged descriptors: Baicalein samples multiple pocket-associated configurations, but none of the descriptor-supported states assemble the canonical AF-2 anchor network.

The short top-pose sensitivity screen extends this interpretation beyond the single selected docking pose used for production. Although the three 30 ns screens showed pose-dependent local packing, all retained pocket association and none formed a canonical Ser289/His323/His449/Tyr473 AF-2 anchor hydrogen-bond. Thus, the absence of persistent canonical AF-2 anchoring was not readily explained by one idiosyncratic selected docking pose. This finding does not remove the selected-pose limitation entirely, because the alternative poses were evaluated by short screens rather than independent full-length production replicas (Koes et al., 2013; Seeliger and de Groot, 2010). It does, however, narrow the most direct pose-selection concern: alternative high-ranking pocket-localized starting poses did not rapidly relax into an anchor-positive state under the same descriptor criteria.

The chemistry-state sensitivity screens directly address the main limitation of the original neutral baicalein/HIE323–HIE449 model. A 7-O monoanionic baicalein model retained pocket association and did not recover persistent AF-2 anchoring, and a neutral baicalein/HID323–HID449 model similarly remained anchor-negative under the same AF-2 hydrogen-bond criteria. These results support the robustness of the anchor-negative interpretation across the tested chemistry states while retaining the chemistry-state boundary of the model. They do not exhaustively sample all possible ligand-protonation, phenolic tautomer, histidine charge-state, or long-timescale transition conditions, and therefore the conclusion remains bounded to the tested short screening conditions.

### 4.5 Functional interpretation and inference boundaries

The recent T2DOP study provides an external functional context for baicalein as a PPARγ-associated modulator in bone–lipid imbalance, including *in vivo* effects on bone microarchitecture and marrow adiposity; *in vitro* effects on bone marrow mesenchymal stem cells (BMSC) osteogenic/adipogenic differentiation; and PPARγ–LBD engagement supported by docking, CETSA, and MD analyses (Zhang et al., 2026). The present simulations address a narrower structural question. They test whether the selected docking-derived baicalein–PPARγ–LBD model forms the canonical AF-2 anchor geometry under the sampled conditions. Therefore, the anchor-negative ensemble proposed here should be interpreted as a structural model adjacent to the reported functional calibration of PPARγ, not as direct proof of transcriptional recalibration, osteogenic rescue, or anti-adipogenic activity.

Functionally, the simulations support a restricted structural interpretation (Ahmadian et al., 2013; Tontonoz and Spiegelman, 2008; Choi et al., 2010; Choi et al., 2011; Zhang et al., 2026; Zhang et al., 2022). Baicalein can remain pocket-associated in the PPARγ ligand-binding pocket while maintaining local H12 order and mixed arm–region interactions without forming the canonical AF-2 anchor hydrogen-bond network. This pattern is compatible with non-canonical pocket engagement, but it is not a direct demonstration of transcriptional activity, partial agonism, or altered receptor signaling.

Several boundaries are important. First, H12 α-helicity, helix-like content, backbone hydrogen-bond persistence, and axial order are structural descriptors (Kroker and Bruning, 2015; Bruning et al., 2007; Hughes et al., 2012; Hughes et al., 2014; Chrisman et al., 2018; Shang et al., 2019; Shang et al., 2020). They do not by themselves prove AF-2 functional activation. Second, MM/GBSA decomposition was used descriptively to compare stabilizing interaction patterns across arms, not to rank absolute binding affinities (Hou et al., 2011; Valdés-Tresanco et al., 2021). Third, the Tyr473 PMF was a local coordinate-specific analysis and does not quantify global binding free energy or pocket thermodynamics (Ngo and Pham, 2022). Fourth, the absence of AF-2 anchor hydrogen bonds is defined within the sampled trajectories and the specified geometric criteria. It is a consistent negative signal within this computational framework, but it does not exclude rare or unsampled baicalein conformations under other conditions.

Within these limits, the selected docking-derived baicalein model is best described as a pocket-associated, measurably H12-organized, anchor-negative bound ensemble with replica-dependent contact persistence. Its stabilization is carried by mixed Arms II and III engagement and residue-level packing contacts, whereas the canonical Ser289/His323/His449/Tyr473 anchor assembly remains absent in the analyzed simulations. Future longer-replica, full-length alternative-pose, broader chemistry-state simulations, and direct experimental structural or biophysical measurements would be needed before extending this selected-pose model to a definitive baicalein binding mechanism.

## Data Availability

The original contributions presented in the study are included in the article/Supplementary Material; further inquiries can be directed to the corresponding authors.

## Supplementary material

SUPPLEMENTARY FIGURE S1 Replica-resolved ligand-retention QC traces for baicalein. The plotted guides follow the bound-state criteria used in Methods and Supplementary Table S1: minimum-distance ≤0.35 nm, contact-count ≥5, and COM distance ≤3.0 nm, with ligand RMSD shown only as a pose diagnostic. These QC traces provide the full retention context summarized in Figure 1A

SUPPLEMENTARY FIGURE S2 Global protein-level QC summaries for the analyzed systems. Protein backbone RMSD, radius of gyration, and solvent-accessible surface area traces were used as trajectory-level stability diagnostics and were not substituted for ligand-bound-state filtering.

SUPPLEMENTARY FIGURE S3 Helix 12 (H12) diagnostic traces and orthogonal local-order metrics. DSSP-derived H12 α-helical and helix-like fractions, backbone hydrogen-bond persistence, RMSF, and axial order descriptors were used to test whether H12-order classification depended on a single secondary-structure metric.

SUPPLEMENTARY FIGURE S4 MM/GBSA convergence diagnostics. Replica-level endpoint-energy convergence was evaluated with block consistency, effective sample size, lag-1 autocorrelation, confidence and bootstrap intervals, and trimmed-mean stability. These diagnostics bounded the use of MM/GBSA as descriptive arm-resolved energetic information rather than absolute binding-affinity ranking.

SUPPLEMENTARY FIGURE S5 Baicalein ProLIF interaction fingerprints. The supplementary heat map reports residue-level interaction-class occupancies computed from the bound-state-filtered 10–90 ns frame set by replica, the consensus panel provides a consensus interaction summary, and the interaction-type summary aggregates ProLIF classes across the three replicas. These fingerprint occupancies are residue-level geometric interaction descriptors and are distinct from the activation function-2 (AF-2) direct hydrogen-bond analysis.

SUPPLEMENTARY FIGURE S6 PMF window-quality diagnostics for the local Tyr473-engagement coordinate. The figure summarizes window overlap and bootstrap uncertainty for the reported PMF profiles; non-converged preliminary tests were retained only as descriptive caveats and were not used for mechanistic inference.

SUPPLEMENTARY FIGURE S7 Descriptor-support analysis for exploratory state annotation. The figure reports state assignments, descriptor availability after replica- and time-matched integration, match fractions, and interpretation boundaries for target-system macrostates. For baicalein, states A–C were included but interpreted conservatively because descriptor-support was limited.

SUPPLEMENTARY FIGURE S8 Atlas robustness controls. Bootstrap and leave-one-replica summaries evaluate whether the reference-state atlas placement and target-system descriptor classification were sensitive to individual replicas or sampling perturbations.

SUPPLEMENTARY FIGURE S9 Sensitivity analyses for the baicalein model. Panel A shows a descriptor PCA generated after excluding Ser289 HB, Tyr473 HB, and activation function-2 (AF-2) anchor-union features from the PCA input. Panel B summarizes all-replica versus replica 1 + replica 2 sensitivity after excluding replica 3. AF-2 anchor descriptors remained zero, whereas contact retention and the normalized Arm I MM/GBSA descriptor were more replica sensitive. Panel C reports descriptor-match fractions for Figure 7 state annotation, and Panel D summarizes the corresponding interpretation boundary.

SUPPLEMENTARY FIGURE S10 Short top-pose sensitivity screening of baicalein docking poses. Panel A shows the three baicalein docking-derived starting poses used for short screening: the selected docking pose used for production and two alternative high-ranking pocket-localized poses. Panel B reports the bound-state retained fraction in the 10–30 ns analysis window after applying the same bound-state criteria used for the production analysis. Panel C reports the strict activation function-2 (AF-2) anchor hydrogen-bond union occupancy across Ser289, His323, His449, and Tyr473; all three screens remained anchor-negative. Panel D shows the Tyr473 hydroxyl-to-ligand acceptor distance distributions, indicating displaced Tyr473 engagement across the three starting poses. These screens test the immediate starting-pose dependence of the negative AF-2 anchor signal and are not full production-replica replacements.

SUPPLEMENTARY FIGURE S11 Protonation and His323/His449-tautomer sensitivity screening for baicalein. **(A)** Chemistry states tested in the sensitivity analysis: the original neutral baicalein/HIE323–HIE449 reference model, 7-O monoanionic baicalein with HIE323–HIE449, and neutral baicalein with HID323–HID449. The neutral/HIE reference corresponds to the original 3 × 100 ns production replicas, whereas the two chemistry variants were evaluated in 3 × 50 ns screening simulations. **(B)** Pocket-retained frame fractions under the same ligand-retention criteria used in the production analysis. **(C)** activation function-2 (AF-2) anchor-union hydrogen-bond occupancy across Ser289, His323, His449, and Tyr473. AF-2 anchor-union occupancy remained ≤0.05% across all sensitivity replicas, indicating that persistent canonical AF-2 anchoring was not recovered. **(D)** Tyr473 hydroxyl-to-ligand acceptor distance distributions for the two chemistry sensitivity variants. These simulations were used as short chemistry-state sensitivity screens and were not treated as full production-replica replacements.

SUPPLEMENTARY TABLE S1 Bound-state and ligand-retention QC summary. The table reports the ligand-retention quality control metrics used to define bound-state-filtered frames, including retained and excursion fractions; pass fractions for the minimum-distance, contact-count, and COM criteria; and ligand RMSD as a pose-orientation diagnostic.

SUPPLEMENTARY TABLE S2 MM/GBSA convergence diagnostics. The table summarizes per replica endpoint-energy convergence diagnostics, including block consistency, effective sample size, confidence intervals, autocorrelation, bootstrap support, and trimmed-mean stability used to bound MM/GBSA interpretation.

SUPPLEMENTARY TABLE S3 Local Tyr473-engagement PMF summary. The table reports numerical summaries of the local Tyr473 hydroxyl-to-ligand-acceptor PMF profiles, including PMF minima and selected coordinate-dependent energy differences in kJ mol^−1^. Values are interpreted as local engagement descriptors, not global binding free energies.

SUPPLEMENTARY TABLE S4 Exploratory coarse graining summary. The table reports feature blocks, lag times, retained dimensions, macrostate assignment summaries, and Chapman–Kolmogorov diagnostic values used to describe exploratory state partitioning. These values are model-quality descriptors, not definitive kinetic rates.

SUPPLEMENTARY TABLE S5 Full arm-resolved MM/GBSA decomposition. The table provides replica-resolved Arms I, II, and III stabilizing magnitudes and normalized stabilizing fractions from the endpoint-energy analysis. Fractions are MM/GBSA-derived energetic descriptors and should not be read as contact occupancies.

SUPPLEMENTARY TABLE S6 Short top-pose sensitivity screening summary. The table summarizes the 30 ns sensitivity screen for the selected docking pose and two alternative high-ranking pocket-localized poses, including retained fractions, activation function-2 (AF-2) anchor hydrogen-bond occupancies, AF-2 union occupancy, and Tyr473–ligand acceptor distance summaries.

SUPPLEMENTARY TABLE S7 Protonation and His323/His449-tautomer sensitivity screening summary. The table summarizes 50 ns sensitivity screens for 7-O monoanionic baicalein under the HIE323/HIE449 model and neutral baicalein under the HID323/HID449 model. Reported quantities include retained fraction; activation function-2 (AF-2) anchor hydrogen-bond occupancies for Ser289, His323, His449, and Tyr473; AF-2 anchor-union occupancy; Tyr473 hydrogen-bond occupancy; Tyr473–ligand acceptor distance summaries; and interpretation flags. These simulations were used as short chemistry-state sensitivity screens and were not treated as full production-replica replacements.
